# Reduced Frontal Nogo-N2 With Uncompromised Response Inhibition During Transcutaneous Vagus Nerve Stimulation—More Efficient Cognitive Control?

**DOI:** 10.3389/fnhum.2020.561780

**Published:** 2020-10-06

**Authors:** Mia Pihlaja, Laura Failla, Jari Peräkylä, Kaisa M. Hartikainen

**Affiliations:** ^1^Behavioral Neurology Research Unit, Tampere University Hospital, Tampere, Finland; ^2^Faculty of Medicine and Health Technology, Tampere University, Tampere, Finland; ^3^Faculty of Biological and Environmental Sciences, University of Helsinki, Helsinki, Finland

**Keywords:** tVNS, transcutaneous vagus nerve stimulation, neuromodulation, cognition, cognitive control, executive function, ERP, N2

## Abstract

We have previously shown invasive vagus nerve stimulation to improve attention and working memory and alter emotion-attention interaction in patients with refractory epilepsy, suggesting that VNS might be useful in the treatment of cognitive impairment. The current research focuses on whether non-invasive, transcutaneous vagus nerve stimulation (tVNS) has similar effects to VNS. Furthermore, we aimed to assess whether tVNS has an impact on cognitive control in general or on underlying brain physiology in a task that mimics everyday life demands where multiple executive functions are engaged while encountering intervening emotional stimuli. Event-related potentials (ERP) evoked in such a task, specifically centro-parietal P3 and frontal N2 were used as biomarkers for attention allocation and cognitive control required to carry out the task. A single-blinded, sham-controlled, within-subject study on healthy subjects (*n* = 25) was conducted using Executive Reaction Time Test (RT-test), a Go/NoGo task engaging multiple executive functions along with intervening threat-related distractors while EEG was recorded. tVNS at the left tragus and sham stimulation at the left ear lobe was alternately delivered throughout the task. To assess the impact of tVNS on neural activity underlying attention and cognitive control, centro-parietal P3 and frontal N2 peak amplitudes were measured in Go and NoGo conditions. Task performance was assessed with RTs and different error types reflecting cognitive control in general and distinct executive functions, such as working memory and response inhibition.No significant effects due to tVNS on performance in the Executive RT-test were observed. For N2 there was a main effect of stimulator status and a significant interaction of trial type (Go, NoGo) and stimulator status. *Post hoc* analysis revealed that tVNS resulted in a significant reduction of frontal N2 only in the NoGo condition. No significant effects were observed for P3 nor were there any effects of emotion. Diminished NoGo-N2 potential along with unaltered task performance during tVNS suggests fewer cognitive control resources were required to successfully withhold a prepotent response. Though caution is warranted, we suggest that tVNS may lead to more efficient neural processing with fewer resources needed for successful cognitive control, providing promise for its potential use in cognitive enhancement.

## Introduction

While cognitive impairment, specifically executive dysfunction, is a frequent consequence of many brain disorders, such as brain damage (Riepe et al., 2004), epilepsy (Holmes, 2015), depression (Rogers et al., 2004), and conditions like burnout (Deligkaris et al., 2014), adequate therapies are lacking. To find new therapies for brain disorders and cognitive dysfunction, a growing field of research is investigating the impact of different neuromodulation techniques on brain functions. Vagus nerve stimulation (VNS) is an invasive neuromodulation technique used for the treatment of pharmacoresistant depression (O’Reardon et al., 2006) and refractory epilepsy (Englot et al., 2015). We have recently shown invasive VNS to have beneficial effects on human executive functions, specifically working memory (Sun et al., 2017b), suggesting that VNS might be useful in the treatment of cognitive impairment. Considering the costs and risks involved in invasive stimulation, it is of great interest to determine whether a safer non-invasive stimulation, transcutaneous vagus nerve stimulation (tVNS; Badran et al., 2018b; Redgrave et al., 2018), has similar potential for enhancing executive functions.

The increased noradrenergic activity has been suggested as a potential mechanism of cognitive enhancement due to VNS. Executive functions rely heavily on noradrenergic transmission and increased noradrenaline (NA) levels have been shown to improve executive functions in rodents (Roosevelt et al., 2006; Grimonprez et al., 2015b), and to improve performance in human clinical populations with deficits in executive functions (Neuhaus et al., 2007; De Taeye et al., 2014). The neural effects of tVNS and VNS are thought to be based on the anatomical connections of the vagus nerve (VN) to brain stem nuclei releasing neurotransmitters such as NA in many brain regions *via* widespread connections (Fanselow, 2012; Butt et al., 2019). tVNS excites the afferent fibers of the auricular branch of the VN in the ear while VNS excites the cervical trunk of the VN. Stimulation is thought to propagate *via* VN to the brainstem nuclei, most importantly nucleus tractus solitarius (NTS), dorsal raphe nuclei (DRN), and locus coeruleus (LC; Van Bockstaele et al., 1999), which is the major source of NA in the brain (Aston-Jones and Cohen, 2005). Functional magnetic resonance imaging (fMRI) studies have shown activation of these brain areas and many others due to tVNS (Kraus et al., 2007, 2013; Dietrich et al., 2008; Frangos and Komisaruk, 2017).

However, there is only partial evidence for the tVNS activating LC-NA system. In human studies, indirect markers of noradrenergic activation in the brain such as pupil size, salivary alpha-amylase (sAA) level, and P3 event-related potential (ERP) amplitude have been used to assess the impact of neuromodulation, such as tVNS and VNS, on NA release with only some markers pointing towards increased NA (Colzato and Beste, 2020). sAA level has been observed to increase due to tVNS (Ventura-Bort et al., 2018; Warren et al., 2019). In contrast, no effect on pupil size has been observed in tVNS studies on healthy subjects (Keute et al., 2019; Warren et al., 2019; Burger et al., 2020). In VNS studies on patients with epilepsy and major depression pupil diameter enlargement has been observed in one study (Jodoin et al., 2015), but not in another one (Schevernels et al., 2016). P3, on the other hand, is thought to be modulated by the phasic response of LC-NA system (Polich, 2007; Nieuwenhuis et al., 2011) and has been suggested as a potential biomarker for the efficacy of VNS in patients suffering from epilepsy (De Taeye et al., 2014). However, the results on P3 amplitude due to tVNS are mixed (Fischer et al., 2018; Ventura-Bort et al., 2018; Warren et al., 2019) and impact of tVNS on P3 may only be found in very specific circumstances (Warren et al., 2020). In addition to the claimed increase in NA release, VNS and tVNS may increase GABA release (Capone et al., 2015; Colzato and Beste, 2020) and long term stimulation may also lead to increased serotonin levels (Dorr and Debonnel, 2006). Increased GABA release could support cortical inhibitory control processes (Hermans et al., 2018) and better signal-to-noise ratio (Leventhal et al., 2003) like NA (Woodward et al., 1979; Hirata et al., 2006), while serotonin has been linked to impulse control and cognitive flexibility (Clarke et al., 2007; Duke et al., 2013).

Even though the underlying mechanism of action of tVNS remains elusive (see recent review by Colzato and Beste, 2020), multiple studies are showing positive effects of tVNS on cognitive functions. Wide range of cognitive functions including associative memory (Jacobs et al., 2015), attentional processing (Ventura-Bort et al., 2018), response selection (Jongkees et al., 2018), adaptation to conflict (Fischer et al., 2018), speech category learning (Llanos et al., 2020) and working memory dependent inhibitory control processes (Beste et al., 2016) have been shown to improve due to tVNS. Beste et al. (2016) suggested that enhancement of inhibitory control, when working memory was involved in the task, was probably caused by the improved signal-to-noise ratio, which has been associated with the functioning of the LC-NA system (Woodward et al., 1979; Hirata et al., 2006).

Executive functions, such as working memory, response inhibition, and set-shifting, are higher-order cognitive control processes that allow for goals rather than impulses or habits to guide behavior (Miyake et al., 2000; Diamond, 2013). For example, cognitive control is needed for overriding prepotent responses, ignoring things irrelevant to the current goals (distractors), and for the ability to flexibly shift to novel behaviors when old habits and impulses are uncalled for. Thus, efficient cognitive control is crucial for successful daily living and on the other hand, executive dysfunction is frequently more detrimental to effective functioning in the society than dysfunction in other cognitive domains (Back-Madruga et al., 2002; Marshall et al., 2011). Considering that many brain disorders involving the prefrontal cortex or its widespread circuits result in executive dysfunction and that executive functions reflect brain health in general (Diamond, 2013; Jacobs et al., 2013; Erkkilä et al., 2018), accurate, sensitive, and ecologically valid assessment methods are crucial (Lezak, 1982).

Even when interfering with everyday life, executive dysfunction may not always be depicted in conventional neuropsychological tests, such as the Stroop test, the Tower of London test, or the Wisconsin Card Sorting Test (Hanna-Pladdy, 2007; Verdejo-García and Pérez-García, 2007; Løvstad et al., 2012a). Conventional neuropsychological tests are performed in emotionally neutral and structured environments, as opposed to unstructured and emotionally burdening everyday life situations that require a far greater extent of cognitive control (Chaytor and Schmitter-Edgecombe, 2003; Kuusinen et al., 2018). Consider the vastly different requirements for cognitive control in a quiet testing room, where emotionally supportive neuropsychologist gives clear instructions what to do and one can fully focus on a single task at hand, in contrast to a busy office with multiple tasks, unexpected interruptions and occasional emotional events, such as a frustrated co-worker giving unpleasant feedback.

Emotional distractors, especially when threat-related, tend to compete for the same attentional and cognitive control resources as the ongoing task, impairing the task performance that relies on executive functions and attention allocation to the task-relevant stimuli (Hartikainen et al., 2000, 2007; Hartikainen et al., 2010a, 2012). Additional cognitive control is therefore required in the context of emotional distractors to maintain attention at the task-relevant stimuli and to control for the extent of automatic emotion-related responses. Based on clinical knowledge, the everyday situations where cognitive control typically fails in individuals with executive dysfunction tend to involve an emotional component. To that end, when mimicking everyday requirements for cognitive control, adding an emotional distractor to the task is called for.

Standardized neuropsychological tests of executive functions are linked with substantial learning effect in repeated testing, limiting their reliable use in the assessment of the impact of an intervention, such as non-invasive neuromodulation, on executive functions (Bartels et al., 2010). Haatveit et al. (2015) suggested that even a relatively long testing interval, such as one year, is not long enough to eliminate learning effects in tests requiring inhibition and mental flexibility. Also, temporal accuracy in conventional pen and paper tests is within a range of seconds limiting sensitivity. In contrast, computer-based reaction time tests of executive function, such as Executive Reaction Time-Test (Executive RT-test; Hartikainen et al., 2010b) used in the current study, allow measurement within the temporal range of rapid mental events, i.e., milliseconds, and is relatively robust to learning effect (Erkkilä et al., 2018). Executive RT-test is an integrated test of working memory, shifting, response inhibition, and emotional control, that engages frontal circuits diversely by engaging multiple executive functions simultaneously in the context of task-irrelevant, threat-related emotional stimuli (Hartikainen et al., 2010b). Performance speed in the test has been shown to correlate with the subjective evaluation of executive functions in daily life (Erkkilä et al., 2018), suggesting this experimental test succeeds in mimicking everyday life demands for cognitive control better than conventional tests, which typically measure one isolated function at the time (Alvarez and Emory, 2006) and which tend to show poor correlation with subjective reports of executive dysfunction in daily life (Løvstad et al., 2012b).

Executive RT-test is sensitive to subtle alterations, both impairment, and improvement, in executive functions and emotion-attention interaction due to neuromodulation (Hartikainen et al., 2014; Sun et al., 2015, 2016, 2017b), cardiac surgery (Liimatainen et al., 2016), and brain injury (Mäki-Marttunen et al., 2015, 2017; Kuusinen et al., 2018). The evidence for Executive RT-test truly assessing executive functions and the underlying fronto-thalamic circuits have been obtained with invasive deep brain stimulation (DBS) studies. DBS allows for temporarily disrupting the functioning of key nodes in these circuits (Hartikainen et al., 2014; Sun et al., 2015; Peräkylä et al., 2017). Disruption of specific executive functions linked with spesific fronto-thalamic circuits (Watanabe and Funahashi, 2012) was found using "lesion on-demand" method with the Executive RT-test (Peräkylä et al., 2017). Thus, in addition to reflecting the subjective assessment of one’s executive functions in daily life, sensitivity to subtle alterations in executive functions in different clinical populations and robustness in repeated testing, Executive RT-test is sensitive to changes caused by neuromodulation in the functioning of fronto-thalamic circuits, which are important for the executive functions (Hartikainen et al., 2014; Sun et al., 2015, 2017b; Peräkylä et al., 2017; Sun et al., 2017a).

In the current study, in addition to investigating the impact of tVNS on cognitive control in general and on specific executive functions, such as working memory and response inhibition, we assessed the impact of tVNS on brain physiology with ERPs. In contrast to behavioral measures, EEG is a direct and sensitive measure of brain function. In contrast to functional imaging studies or conventional neuropsychological assessments, it has a temporal resolution within milliseconds in line with rapid mental functions. Furthermore, EEG allows information to be obtained from alterations in brain functions even when no behavioral response is associated with the task, such as in NoGo condition when the subject is supposed to withhold from responding. Thus, the subtle impact of neuromodulation might be depicted only with ERPs (Sun et al., 2016).

The current study focused on frontal N2 and a subsequent, centro-parietal P3 ERPs. These ERPs are linked to attention and cognitive control, with demand for cognitive control mainly reflected in frontal N2 amplitude (Folstein and Van Petten, 2008), while attentional resources allocated to processing motivationally relevant stimuli are reflected in centro-parietal P3 (Kok, 2001; Polich, 2007). P3 is one of the most studied ERP peaks and it is connected to a variety of cognitive processes and widely distributed brain networks (Downar et al., 2000). Centro-parietal P3, also referred to as P3b, is known to be modulated by several factors including task relevance, emotional relevance, stimulus probability, and LC-NA system activation (Polich, 2007; Nieuwenhuis et al., 2011). P3 amplitude has been reported to both diminish and increase due to noradrenergic activity (De Rover et al., 2015). Previous studies using NoGo-task have suggested that N2 and P3 reflect different phases of response inhibition or response conflict (Falkenstein et al., 1999; Donkers and van Boxtel, 2004), but N2 seems to be a more specific indicator of cognitive control (Kopp et al., 1996; Kraiser et al., 2006) because frontal N2 amplitude has been observed to increase when demand for cognitive control increases (Nieuwenhuis et al., 2003). Donkers and van Boxtel (2004) suggested that NoGo-N2 could be related to conflict monitoring rather than response inhibition as its amplitude increases when the relative frequency of Go responses is significantly higher than NoGo responses. However, the fact that N2 may be larger for NoGo condition even when the conditions are equally frequent, which is the case in the current study, speaks against mere conflict monitoring behind N2 (Lavric et al., 2004).

Only a few studies are reporting the impact of tVNS on ERPs. To our knowledge, there is only one previous report of the impact of tVNS on N2 amplitude (Fischer et al., 2018). Fischer et al. (2018) observed a greater decrement of N2 amplitude after a conflict due to tVNS. The authors suggested that the decrement could be caused by enhancement in the conflict-triggered adaptation of cognitive control due to tVNS. Furthermore, a reduction of N2 amplitude due to noninvasive brain stimulation along with improved cognitive control has been previously reported in a study by Dubreuil-Vall et al. (2019) using frontal transcranial direct current stimulation (tDCS). They speculated based on signal detection theory, that N2 potential reflects external variables (noise) and internal variables (effort) so that lower amplitude reflects the better signal to noise ratio, less effort, and more efficient use of cognitive resources (Dubreuil-Vall et al., 2019). P3, on the other hand, has been used in previous tVNS studies as a biomarker of LC-NA system activation with so far mixed results (Fischer et al., 2018; Ventura-Bort et al., 2018; Warren et al., 2019, 2020).

The current study aimed to find out whether tVNS has an impact on cognitive control as reflected in RTs and error rates in the Executive RT test and/or ERPs; frontal N2 or centro-parietal P3. Furthermore, we wanted to assess whether tVNS has similar effects on executive functions, in particular working memory and on affective responses, as observed with invasive VNS in our previous study (Sun et al., 2017b). We expected the impact of stimulation on neural processing underlying cognitive control to be reflected in frontal N2 amplitude. Assuming tVNS, like VNS, activate the LC-NA system, this would lead to a more efficient neural processing and consequently either improved cognitive performance or less cognitive control resources required to achieve the same performance level. In the latter case diminished N2 might be expected during tVNS in comparison to sham stimulation since frontal N2 amplitude is assumed to reflect the amount of cognitive control resources required to carry out the task. With NoGo condition requiring more cognitive control and typically evoking larger N2, we expected to see the potential effect of tVNS, especially in NoGo-N2. Also, more efficient cognitive control could result in shorter reaction times and fewer commission errors during tVNS. With centro-parietal P3 amplitude intricately linked to attention and the LC-NA system, we assessed whether tVNS alters the centro-parietal P3 amplitude. We had no *a-priori* expectation on the impact of tVNS on P3 amplitude. Thus, ERP findings are interpreted in the context of behavioral findings, i.e., whether improvement, decrement, or no effect on performance is observed.

## Materials and Methods

### Participants

Twenty-five young healthy right-handed subjects, 16 females and nine males (average age 25.5 ± 4.8 years) recruited from the local university participated in the study. The average years of education were 15.3 ± 1.6 years. The sample size was based on simulation-based power analysis with reaction times, showing that with 25 subjects and alpha level 0.05, we have 80% power to detect a difference of 20 ms in reaction time (RT), which is slightly more than the detected difference in the previous VNS study (Sun et al., 2017b) with smaller sample size. The Behavioral Rating Inventory of Executive Functions–Adult version (BRIEF-A; Roth et al., 2005) and the Beck’s Depression Inventory (BDI; Beck et al., 1996) questionnaires were administered to all participants before testing to rule out underlying depression or executive dysfunction. Somatic health problems and medications were reviewed before testing. Exclusion criteria included any history of psychiatric, neurological, cardiac disorder, or abnormal BDI or BRIEF-A result. Participants using any medications impacting the central nervous system (including medication for psychiatric purposes) were excluded. EEG data of seven subjects were excluded from ERP analysis, due to technical difficulties and low signal quality, but their behavioral results were included in the analysis.

### Executive Reaction Time Test (Executive RT-Test)

**Figure 1:**
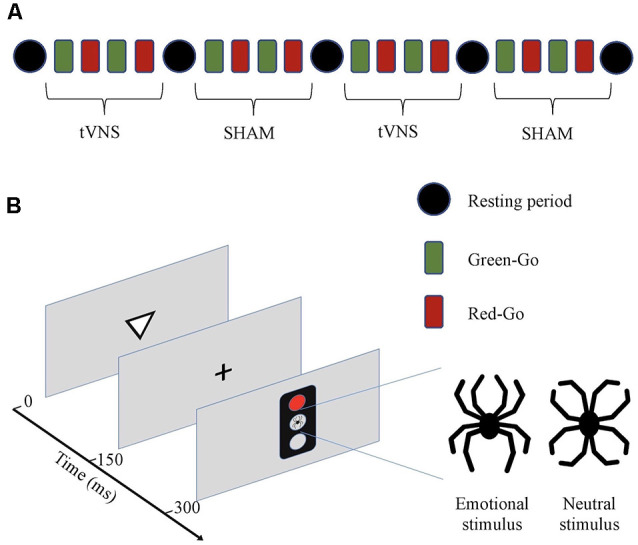
**(A)** Schematic presentation of the experiment. The experiment consisted of two transcutaneous vagus nerve stimulation (tVNS) and two sham stimulation periods. Each period consisted of four blocks of trials with different response rules (Green-Go and Red-Go). Half of the subjects started with tVNS and the other half with sham stimulation. Stimulation type was alternated after every four blocks (10 min) and between different stimulation types, there was a resting period (4 min). **(B)** Executive RT-test (Hartikainen et al., 2010b). Subjects were required to respond to a visual stimulus, a triangle pointing up or down, by pressing as fast and as accurately as possible one of the two keys on a response pad according to the orientation of the previously presented triangle in a Go-condition or withhold from responding in a NoGo-condition. The orientation of the triangle was randomized within each block. Whether the subject was required to respond (Go) to or withhold from responding (NoGo), was indicated by a green or red traffic light. At the beginning of each block, there are instructions as to whether red color indicates a Go (Red-Go) and green a NoGo, or vice versa (Green-Go). The rule for responding changed after each block. A trial begins with a triangle pointing up or down for 150 ms followed by a fixation cross for another 150 ms. Subsequently, a traffic light appears for 150 ms. In the center of the traffic light, there was an emotional stimulus, a black line drawing resembling a spider, or an emotionally neutral control figure resembling a flower, both composed of identical line elements in a different configuration. Total error rate and mean RTs reflect the subject’s cognitive control in general and each error type a specific cognitive function with commission errors reflecting response inhibition, missing responses attention, and incorrect responses working memory, correspondingly.

EEG was recorded while subjects performed the Executive RT-Test, a Go/NoGo test tapping into working memory, response inhibition, emotional interference, and attention (Hartikainen et al., 2010b). See Figure 1 for the schematic presentation of the entire experiment and the Executive-RT-test. RTs and total errors allow assessment of efficiency of cognitive control in general while analyzing different error types allows the assessment of distinct executive functions such as working memory and response inhibition.

Four practice blocks were used to allow for the subject’s performance to stabilize before the start of the actual experiment since it has previously been shown that most of the learning in this task occurs during the first four blocks (Erkkilä et al., 2018). After the practice there were 4 × 4 experimental blocks with half of the blocks during tVNS stimulation and a half during sham stimulation, totaling up to 2 × 4 blocks for each stimulation type and within a stimulation type four blocks with each rule (green-Go vs. red-Go). One subject performed a total of 1,024 trials, i.e., 512 Go- and 512 NoGo-trials, 256 Go- and NoGo-trials during each stimulation type, out of which there were 128 Go and NoGo trials in the context of each distractor type (emotional or neutral) and further divided by Go-NoGo-rule there were 64 trials for each trial type. The impact of learning and fatigue on performance was controlled for with counterbalanced order of sham stimulation and tVNS between subjects. After every four blocks, there was a 4-min resting period when the subject was asked to rest quietly eyes closed and the stimulation type was changed for the next four blocks.

Performance measures in the Executive RT-test include RT and three different error types: incorrect responses, missing responses, and commission errors. In a Go-trial subject can make an incorrect response, i.e., press an incorrect button, or miss responding, reflecting lapses in working memory or attention, correspondingly. In a NoGo-trial a keypress, i.e., a commission error, reflects a failure in response inhibition. Different error types were summed up as total errors indexing cognitive control in general along with RTs.

### Transcutaneous Vagus Nerve Stimulation

tVNS was applied using a CE approved tVNS device (Salustim Group, Kempele, Finland). The device has two ear-clip electrodes. The active stimulation electrode was located in the sensory area of the auricular branch of the vagus nerve in the left ear, the inner surface of the tragus (tVNS), and the sham stimulation electrode was placed on the ear lobe of the same ear, where there are no vagal nerve branches (sham). Before attaching clips, skin on the tragus and earlobe was cleaned and gel used in the EEG was applied to decrease the resistance of the skin under the electrode. The intensity of the stimulation was increased individually in a staircase manner level by level until the subject detected slight tingling in the active electrode. The current was then calibrated to the minimum perceptual level as has been done in an fMRI study showing activation of cerebral afferents of the vagal pathway (Badran et al., 2018a). The device has 10 different intensity levels, with levels 2–4 used in the current study. The same intensity level was used for active and sham stimulation. The exact output current for each subject could not be assessed due to differences in subjects’ skin resistance, but at levels 2–4, the maximum output current is between 1.6 mA and 3.2 mA when skin resistance is 5 kΩ. The stimulation mode was constant with a symmetric, biphasic rectangular waveform, 250 μs pulse width, and 30 Hz pulse rate, the same frequency as used in the study of Sun et al. (2017b).

The experiment was conducted as a single-blinded placebo-controlled within-subject study. The subjects were not aware of the differences in active vs. sham stimulation locations or any hypothesis related to the study. Every other subject started with active stimulation and every other with sham stimulation. Active and sham stimulation alternated throughout the test so that half of the subjects performed the first and the third blocks under tVNS and the second and the fourth under sham stimulation or the other half the other way around. Recordings were done between 11 am and 5 pm. Subjects did not report any side effects during the test or immediately after it.

### Electroencephalography (EEG) and Data Processing

EEG was recorded using 64 Ag/AgCl active electrodes (actiCAP, Gilching, Germany), QuickAmp amplifier system, and Brain Vision Recorder software (Brain Products, GmbH) at 500 Hz sampling rate. The impedance of all electrodes was kept below 5 kΩ throughout the recordings.

Brain Vision Analyzer software (version 2.1, Brain Products GmbH) was used for the preprocessing of the EEG data and ERP analyses. EEG was re-referenced to linked right and left mastoids and signal was band-pass filtered at 0.1–30 Hz. Blinks and other artifacts were removed using ICA (independent component analysis) based correction. After ICA correction intervals with over 80 μV peak to peak voltage difference were removed from the analysis. EEG was epoched into 2,000 ms segments starting from 200 ms before and 1,800 ms after trial onset.

Remaining single-trial EEG segments were averaged for each condition (Go or NoGo), stimulator status (active or sham) and distractor type (emotional or neutral), resulting in eight different ERP conditions for each subject (In both Go- and NoGo-conditions: neutral active, neutral sham, emotionally active and emotional sham). Based on convention (Luck, 2005; Boudewyn et al., 2018), previous experience on this paradigm and visual inspection of the individual ERP averages minimum of 50 clean EEG epochs were required for each ERP condition for the subject to be included in the analysis. The number of trials in different conditions was comparable within each subject included in the analysis. Each trial began with a triangle followed by a Go/NoGo-signal (traffic light) appearing 300 ms after the trial onset. N2 component after the Go/NoGo signal was identified from average waveforms based on the visual inspection and semiautomatic peak detection based on the timeframes defined by visual inspection. N2 was defined as the most negative peak in the timeframe from 200 ms to 350 ms and P3 as a subsequent positive peak appearing between 300 and 500 ms after the traffic light i.e., 500–650 ms and 600–800 ms from the trial onset, correspondingly. Due to the frontal distribution of the N2 peak (Folstein and Van Petten, 2008), we used frontal channels F1, F2, F3, and F4 to measure N2 peak amplitudes. For P3 we used CP1, CP3, CP2, CP4 channels due to centro-parietal distribution of the P3 reflecting task-related attentional resources and activation of LC-NA-system (Polich, 2007).

### Statistical Analysis

Reaction times and ERP peak amplitudes (N2 and P3) were analyzed using repeated-measures analysis of variance (ANOVA). Data were checked for normality and transformed using Yeo and Johnson (2000) transformation if necessary. “Stimulator status” (active vs. sham) and “emotional valence” (emotional vs. neutral) were used as factors in all analyses. ERP analysis had an additional factor, “trial-type” (Go vs. NoGo). Interaction effects were analyzed further with *post hoc* ANOVAs.

Errors were analyzed with a generalized mixed-effects logistic regression (Dixon, 2008; Jaeger, 2008). Each error type was analyzed using a separate model predicting probability to make that kind of an error. “Subject” was used as random effects predictor and “stimulator status” and “emotional valence” as fixed effects predictors. For logistic regression trial outcomes were dichotomized into binary classes so that for total errors classes were “correct” (correct button press in Go-trial or no response in NoGo trial) or “error” (incorrect or missing button press in Go-trial or any button press in NoGo trial), for incorrect responses “incorrect” (incorrect button press) or “other” (correct or missing button press), for missed responses “miss” (missing button press) or “other” (correct or incorrect button press) and for commission errors “commission error” (a button was pressed in NoGo trial) or “correct” (no button press in NoGo trial).

Statistical analysis was done using R statistics v. 3.1.1 (R Core Team, 2019). Repeated measures ANOVA was done with EZ package v. 4.2-2 (Lawrence, 2016) and regression analysis with lme4 package v. 1.1-10 (Bates et al., 2015).

## Results

### Behavioral Results

Behavioral results can be found in Table 1. Statistical analysis of the behavioral data did not result in any significant difference, i.e., tVNS stimulation or emotional stimulus had no statistically significant effect on reaction times (Table 2) or on any error types (Table 3). Due to too low number of missing responses, it was not possible to analyze them.

**Table 1:** Behavioral results of the Executive Reaction time test.

Stimulation	Distractor	Mean Reaction Time (ms, SD)	Median Total Errors (%, IQR)	Median Incorrect responses (%, IQR)	Median Missing Responses (%, IQR)	Median Commission Errors (%, IQR)
Sham	Neutral	327 (57)	2.3 (3.1)	0.8 (1.6)	0.0 (0.0)	0.8 (1.6)
	Emotional	327 (65)	2.3 (3.1)	0.8 (2.3)	0.0 (0.0)	0.8 (2.3)
tVNS	Neutral	325 (58)	1.6 (2.3)	0.8 (1.6)	0.0 (0.8)	0.8 (1.6)
	Emotional	323 (54)	2.3 (3.1)	0.8 (1.6)	0.0 (0.0)	0.8 (1.6)

*IQR, Interquartile Range; SD, Standard Deviation*.

**Table 2:** ANOVA table of reaction times.

Predictor	*df*_num_	*df*_den_	*F*	*p*	$\eta_{\text{G}}^{2}$
Stimulator status	1	24	0.05	0.825	0.00
Distractor	1	24	1.75	0.199	0.00
Stimulator status × Distractor	1	24	0.45	0.510	0.00

**Table 3:** Odds ratios of error analysis.

Predictor	Total errors	Incorrect responses	Commission errors
Intercept	0.01 (0.01–0.02)	0.01 (0.00–0.01)	0.01 (0.01–0.02)
Stimulator ON	0.92 (0.69–1.22)	0.77 (0.49–1.21)	0.90 (0.61–1.33)
Emotional distractor	1.05 (0.80–1.38)	1.07 (0.71–1.63)	0.98 (0.67–1.43)
Stimulator ON × Emotional distractor	1.05 (0.71–1.55)	1.32 (0.72–2.44)	1.00 (0.58–1.72)

*Missing responses are omitted from the table because there was none*.

### ERP Results

**Figure 2:**
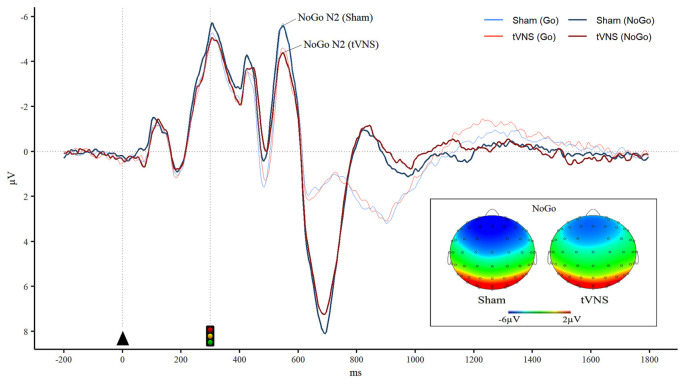
Reduced frontal NoGo-N2 during tVNS. Grand averaged event-related potentials (ERPs) in the frontal region (averaged over F1, F2, F3, and F4 electrodes) illustrates significantly diminished NoGo-N2 ERP peak amplitude during tVNS. Furthermore, brain topography maps in the NoGo condition during tVNS and sham stimulation show reduced frontal negativity in the N2 time window during tVNS in comparison to sham stimulation.

For N2 peak amplitude there was a main effect of stimulator status (tVNS, Sham, *F*_(1,17)_ = 14, 41, *p* = 0.001, $\eta_{\text{G}}^{2}$ = 0.01 and a significant interaction of trial type (Go vs. NoGo) and stimulator status (*F*_(1,17)_ = 5.06, *p* = 0.038, $\eta_{\text{G}}^{2}$ = 0.01 *Post hoc* analysis revealed that tVNS significantly reduced frontal N2 peak amplitude compared to sham stimulation only in NoGo condition: The main effect of repeated measures ANOVA, *F*_(1,17)_ = 17.42, *p* = 0.001, $\eta_{\text{G}}^{2}$ = 0.02, sham = −6.75 ± 4.08 μV, tVNS = −5.73 ± 3.50 μV (Figure 2). Go-N2 did not result in any statistically significant results. There were no significant effects of stimulation on P3 amplitude in either Go or in NoGo condition. There were no effects of emotion on N2 nor P3 amplitudes. N2 and P3 amplitudes are presented in Table 4 and the summary of the statistical analysis in Table 5 and Table 6.

**Table 4:** Mean amplitude (μV) and standard deviation of frontal N2 and centro-parietal P3 peaks during active and sham stimulation.

ERP	Stimulation	Voltage (μV, sd)
N2 Go	Active	−5.73 (5.16)
	Sham	−5.98 (4.83)
N2 NoGo	Active	−5.73 (3.50)
	Sham	−6.75 (4.08)
P3 Go	Active	5.32 (4.13)
	Sham	5.16 (3.69)
P3 NoGo	Active	6.25 (3.43)
	Sham	6.59 (3.36)

**Table 5:** ANOVA table of the frontal N2 peak amplitudes.

N2	*df*_num_	*df*_den_	*F*	*p*	$\eta_{\text{G}}^{2}$
Trial type	1	17	0.37	0.550	0.00
Stimulator status	1	17	14.41	0.001	0.01
Distractors	1	17	0.23	0.635	0.00
Trial type × Stimulator status	1	17	5.06	0.038	0.00
Trial type × Distractor	1	17	0.21	0.651	0.00
Stimulator status × Distractor	1	17	0.99	0.334	0.00
Trial type × Stimulator status	1	17	0.08	0.781	0.00
× Distractor					
**N2 Go**
Stimulator status	1	17	1.81	0.196	0.00
**N2 NoGo**
Stimulator status	1	17	17.42	0.001	0.02

*$\eta_{\text{G}}^{2}$ = Generalized Eta Squared; *df*, degrees of freedom*.

**Table 6:** ANOVA table of the centro-parietal P3 peak amplitudes.

Predictor	*df*_num_	*df*_den_	*F*	*p*	$\eta_{\text{G}}^{2}$
Trial type	1	17	2.20	0.157	0.03
Stimulator status	1	17	0.12	0.738	0.00
Distractors	1	17	0.98	0.337	0.00
Trial type × Stimulator status	1	17	2.40	0.140	0.00
Trial type × Distractor	1	17	1.49	0.239	0.00
Stimulator status × Distractor	1	17	1.20	0.288	0.00
Trial type × Stimulator status	1	17	0.31	0.582	0.00
× Distractor					

*$\eta_{\text{G}}^{2}$ = Generalized Eta Squared; *df*, degrees of freedom*.

## Discussion

The study aimed to investigate whether tVNS has an impact on human cognitive control in healthy subjects at the behavioral or neural level. Furthermore, we assessed whether tVNS improves working memory as previously reported with invasive VNS (Sun et al., 2017b). While no impact of tVNS was found on behavioral measures of attention, working memory, response inhibition, emotional interference, or cognitive control in general, we observed a change in the neural marker of cognitive control, N2 ERP. The peak amplitude of frontal NoGo-N2 was significantly reduced during tVNS in contrast to sham stimulation. Diminished NoGo-N2 amplitude along with uncompromised response inhibition performance suggests that during tVNS less cognitive control resources were required to successfully withhold from responding.

The impact of VNS on enhancing cognitive functions has been previously linked with increased noradrenaline levels in animal studies (Roosevelt et al., 2006; Grimonprez et al., 2015b, see also review Grimonprez et al., 2015a). In human tVNS studies, surrogate markers of noradrenaline levels have been assessed. For example, increased alpha-amylase has been observed following tVNS at the cymba conchae (Ventura-Bort et al., 2018; Warren et al., 2019). Given the connections between the vagus nerve and LC (Kalia and Sullivan, 1982; Van Bockstaele et al., 1999) and the role of the LC-NA system in cognition (Mair et al., 2005; Hirata et al., 2006; Wang et al., 2007; Manta et al., 2013), it is possible that similar to VNS, tVNS may increase the release of noradrenaline. NA is thought to improve the signal-to-noise ratio (Woodward et al., 1979; Hirata et al., 2006), possibly resulting in a more efficient neural processing which would consequently lead to lower resource requirement to complete the task. Another possible neurotransmitter that could potentially contribute to the current findings is GABA as it is thought to be increased by tVNS and believed to contribute to both improved signal to noise ratio and the inhibitory control (Leventhal et al., 2003; Capone et al., 2015; Hermans et al., 2018).

Improved neural processing underlying inhibitory control, or more specifically response inhibition, would lead to a reduced demand for cognitive control resources in a NoGo-task, which would be reflected in reduced NoGo-N2 amplitude. In line with the current results, N2 peak amplitude reduction due to non-invasive neuromodulation, both tDCS, and tVNS, along with enhanced cognitive control or cognitive processes closely linked to it, has been observed in previous studies (Lapenta et al., 2014; Fischer et al., 2018; Dubreuil-Vall et al., 2019). tDCS to the left dorsolateral prefrontal cortex was associated with reduced NoGo-N2 amplitude along with better inhibitory control related to food intake in a study by Lapenta et al. (2014). Similarly, tDCS to the left dorsolateral prefrontal cortex resulted in N2 reduction along with improved cognitive control, as seen in speeded RTs in a Flanker Task in a study by Dubreuil-Vall et al. (2019). In a tVNS study by Fischer et al. (2018), reduction of N2 amplitude was linked with enhanced conflict adaptation. tVNS has also been reported to improve response inhibition when working memory is required (Sellaro et al., 2015). In the current study working memory was required to successfully perform the task, as subjects had to maintain the changing rule of responding and the direction of the previously presented triangle in working memory. Combining response inhibition tasks with a working memory task and intervening emotional distractors competing for attentional resources emphasizes the role of cognitive control in intact task performance. Thus, the reduction of NoGo-N2 in the current study may reflect either more efficient cognitive control in general or more specifically improved inhibitory control in the context of high demand for cognitive control.

In Go/NoGo tasks N2 amplitude is typically greater for the infrequent trial type, even if it is the Go-trial (Nieuwenhuis et al., 2003). In the current study, there was a tendency for NoGo-N2 to be larger than Go-N2 suggesting more cognitive control was required in NoGo condition despite equally frequent Go and NoGo trials. Greater need for cognitive control during NoGo condition probably relates to the rapid presentation of the stimuli as well as task instructions emphasizing speed in an RT test and consequently biasing to Go-responding as suggested by Nieuwenhuis et al. (2003). While there was a main effect of stimulation, the interaction between stimulation and trial type pointed towards the distinct effect of stimulation in NoGo trials where there was a significant reduction of N2 amplitude due to stimulation unlike in Go-trials. NoGo-N2 has been suggested to reflect competition between execution and inhibition of a response rather than response inhibition *per se* linking N2 with cognitive control (Nieuwenhuis et al., 2003). Moreover, ACC, a frontal brain region involved in cognitive control (Løvstad et al., 2012b), has been indicated as one of the neural sources contributing to N2 (Nieuwenhuis et al., 2003). However, other sources differentiate NoGo-N2 from Go-N2 even when both trial types are presented with equal frequency, for example, sources thought to relate more specifically to response inhibition, such as sources localized to the right ventral and dorsolateral prefrontal cortex (Lavric et al., 2004). Thus, N2 potential is not specific to response inhibition or any other cognitive process but reflects many different cognitive processes and activity in many different brain regions with a common denominator in cognitive control functions concentrated in frontal networks.

Though no impact on behavioral measures of cognitive or affective functions was observed due to tVNS, we observed a significant reduction in N2 peak amplitude during a NoGo condition. While impaired or improved response inhibition performance can be observed with increased or decreased commission errors correspondingly, successful response inhibition is not linked with any visible behavioral response. ERPs provide information on cognitive brain functions related to response inhibition and cognitive control even in the absence of behavioral response. Furthermore, ERPs provide a direct and more sensitive measure of the impact of neuromodulation on brain functions than behavior (Sun et al., 2016) providing online information of brain functions while no behavioral changes can be observed (Boly et al., 2011; Kouider et al., 2013).

While a higher number of Go-trials than NoGo-trials makes response inhibition task harder and tends to result in a greater number of commission errors, there are some benefits to having an equal number of both trial types. When there is low-frequency of NoGo trials the brain response evoked by a NoGo trial, such as ACC activation, may reflect responding to a low-frequency event rather than response inhibition *per se* similar to an oddball paradigm, where the low-frequency event is the target linked with a response (Braver et al., 2001). In the current study, there were also other reasons for keeping the frequencies equal. There were several different trial types, two different response rules, and two different stimulation types alternating within the session. An equal number of Go and NoGo trials were justified, to keep the experiment unbiased and symmetrical and to obtain an adequate number of trials for each condition for reliable ERPs in a time frame that is feasible for the subjects to be engaged in an attention task. The signal to noise ratio improves as a function of the square root of the number of trials included in the average, i.e., the more trials included in the average the better (Luck, 2005). However, practical aspects such as the length of the experiment limit the number of trials that can be presented to the subject. Even though the frequencies of the Go and NoGo trials were identical in the current study there was still a need to inhibit responding in a NoGo trial. There was an additional feature to conventional NoGo-paradigms in the Executive RT test, i.e. the rule for responding kept changing throughout the experiment. In the case of a green traffic light indicating a NoGo-rule, the subject had to overcome an overlearned rule where the green traffic light is associated with going and red traffic light with stopping. Furthermore, with the rule for responding changing frequently throughout the experiment, the subject had to withhold from responding according to the previous rule. Thus, even though the 50/50 frequency is not the convention in a typical NoGo-paradigm we are confident the subjects had to engage extra cognitive control to withhold from responding in a NoGo trial with a bias towards responding not due to infrequency of NoGo condition but due to a fast RT-test and either overlearned or previous rule biasing towards responding.

In the previous study by our group (Sun et al., 2017b), invasive VNS was shown to be effective in improving working memory performance in patients with refractory epilepsy. In contrast to that study, in the current study, we did not observe improvement in performance during tVNS in healthy young subjects. In the study conducted by Sun et al. (2017b), baseline cognitive performance of participants was likely compromised due to their epilepsy and medications used to treat it. Also, the task was challenging for the subjects in Sun et al. study. In the current study, the same task was too easy for young, healthy, highly educated subjects, whose error rates were very low leaving no room for improvement (ceiling effect). Even if there was a ceiling effect in errors, in RT there is no ceiling effect and a change in RT could have shown the improvement or the decrement in the cognitive control. However, there was no difference in RT between active and sham stimulation in any condition. In the future, subjects with suboptimal cognitive performance and a challenging enough task should be used to investigate whether tVNS has potential for improving compromised cognitive performance.

While it was important to investigate whether noninvasive tVNS has similar effects on cognitive performance as invasive VNS, it was not surprising that we did not find similar behavioral results with tVNS in healthy subjects as we did with the invasive VNS in a clinical population. In addition to obvious differences in the study groups, it is evident that tVNS applied to a sensory afferent of the auricular branch of the vagus nerve transcutaneously is not as effective neuromodulation method as invasive VNS applied to the cervical trunk of the vagus nerve. Yet, despite its limitations, the current study is instrumental in that identical paradigm was used as in the previous study with invasive VNS, providing invaluable insight into the effectiveness of tVNS in comparison to invasive VNS in enhancing cognitive functions.

The stimulation parameters used in this study were chosen according to the previous VNS study; stimulation frequency of 30 Hz and pulse width of 250 ms were identical in both studies. Instead of a constant level of current across individuals, we used individual minimal perceptual threshold when adjusting stimulation intensity at the beginning of the test similarly to many previous studies (Badran et al., 2018a; Fischer et al., 2018; Keute et al., 2019). While stimulation current varied between subjects, the current was always over 0.5 mA, suggested to be the minimum level to obtain an effect on sAA with tVNS (Ventura-Bort et al., 2018; Warren et al., 2019; Colzato and Beste, 2020). A constant level of current or voltage across individuals does not guarantee a similar impact in the nerve or the brain, thus using a sensory perception to systematically guide titration of the stimulation intensity is justified. Even stimulation intensities given below the perceptual threshold have been shown to evoke reliable brainstem potentials, similar to those evoked by above-threshold stimulation, as well as result in positive cognitive effects such as faster speech category learning (Llanos et al., 2020).

In addition to stimulation intensity, individual baseline neurocognitive state is likely to influence the impact of neuromodulation. The relationship between cognitive performance and the level of a neurotransmitter impacting cognitive control, such as NA, can be illustrated with an inverted U-curve according to Yerkes-Dodson law (Yerkes and Dodson, 1908; Arnsten, 2000, 2011; Berridge and Spencer, 2016). If the NA level is at the top of the inverted U-curve at the baseline before an intervention, such as neuromodulation, no further improvement in performance can be expected. But if the level is suboptimal, an increase in the NA level may lead to an improvement in cognitive performance. Yerkes-Dodson’s principle may be one of the reasons why improvement in performance may not be expected if the neurotransmitter level and cognitive performance are already at the optimal level, as is likely the case with optimally performing young healthy subjects. Similar to the impact of neuromodulation on NA-mediated performance, P3 amplitude depends on the baseline NA-activity (De Rover et al., 2015). Thus, whether VNS, tVNS, or any other neuromodulation method modulates P3 amplitude or cognitive performance will likely depend on the baseline level of neurotransmitter activity and cognitive functions of the study group. Thus, not surprisingly, in the current study, no significant impact of tVNS on P3 amplitude was obtained.

The study had some limitations. Possibly the differences in the N2 amplitude were a result of stimulation artifact contaminating the EEG channels, especially in the reference electrodes located on the mastoid behind the ear. If that was the case though, we should have observed the same reduction in all conditions, and the effect would not have been limited to NoGo-condition only. Furthermore, since identical electrical stimulation was administered during active and sham stimulation only to slightly different locations, any contamination related to the stimulation artifact would likely have been of similar magnitude during active and sham stimulation. Thus, the stimulation artifact is not likely to explain the differences between the two conditions. Additionally, we took several measures to minimize the potential impact of stimulation artifact. All EEG channels were filtered below 30 Hz and we excluded from the analysis subjects whose EEG was contaminated by artifacts or who did not produce clearly distinguishable N2 peaks in the visual inspection, for any reason. This reduced our ERP sample from 25 to 18 subjects. Taken together, it is plausible to conclude that the impact of stimulation on cognitive control functions was a real effect, rather than a confound due to stimulation artifact. However, future studies are needed to confirm this finding and link them to behavioral results. Further, it is not entirely excluded that the decrease in N2 amplitude might be detrimental to performance if sensitive enough performance measures were used. Irrespective of whether the observed change in N2 amplitude reflects beneficial or detrimental effect of tVNS on response inhibition performance, it provides evidence that tVNS has an impact on cognitive brain potentials and the underlying neural circuits opening opportunities for modulating neural circuits.

It is important to note that unlike previous studies on tVNS the current study focused exclusively on the immediate and short-term effects of tVNS on the cognitive control and specific executive functions along with neural factors underlying them. The current study does not address the long-term effects of tVNS on cognition and it may well be that for more prolonged effects on cognition stimulation period needs to be longer as well. No previous study has used active and sham tVNS stimulation in the same session to our knowledge. It is feasible that the lack of observed behavioral effects of tVNS may have been partly due to the temporal design of the study. If the 4 min resting period in between the active and sham stimulation was not enough to wash out the effects of the stimulation, there may have been some lingering effects of tVNS during the immediately following sham stimulation period diluting potential differences between the active and sham stimulation. However, different wash-out periods apply for different effects of tVNS from ms range of nerve cell firing to possibly hours and days related to neuroplasticity. The fastest observed effects of tVNS occur only 2 ms after the stimulation pulse in the brainstem, as evidenced with vagal brainstem evoked potentials (Llanos et al., 2020) which are the most direct measure of vagal activation due to tVNS in humans. While we did not measure vagal brainstem evoked potentials, the counterbalanced sham-controlled within-subject within-session study design that controls for confounding factors allows for inferring the later cognitive control-related ERP effect to be due to vagal activation.

Future studies are needed to explore possible long-term effects of tVNS on cognition and neurophysiology with longer stimulation and wash-out periods. Moreover, cymba conchae have been suggested to be a more efficient location to stimulate vagus nerve compared to the inner surface of the tragus used in the current study (Ellrich, 2019). On the other hand, fMRI studies have shown similar brain effects when stimulating either location (Kraus et al., 2007; Dietrich et al., 2008; Badran et al., 2018a) and according to review by Butt et al. (2019), based on the anatomy of the vagus nerve innervations there is no clear difference between the two locations. However, to assess the potential for enhancing cognitive functions with tVNS, future studies are needed to assess whether the impact of tVNS on brain functions depends on baseline cognitive performance, on the stimulation location, or other stimulation parameters.

As for the strengths of the study, according to our knowledge, this is the first single-blinded, sham-controlled study, where active and sham stimulation were compared within the same subject during the same test session. The within-subject study allows for better control over subject-specific confounding factors influencing cognitive performance and cognitive ERPs than comparing two groups. Similarly, within-session study, where periods of tVNS and sham stimulation alternate allows for controlling multiple factors that alter cognition day to day such as sleep, caffeine intake, arousal, fatigue, motivation level, mood, etc. For example, some tVNS effects on cognition have been observed only in individuals with low positive moods (Steenbergen et al., 2020), emphasizing the benefits of within-subject and within-session approach. The within-subject study also has higher statistical power allowing a smaller sample size. Furthermore, the Executive RT Test, while too easy for our sample, allowed for investigating subtle brain effects of stimulation on different cognitive control processes, such as response inhibition, and a comparison to previous neuromodulation studies, including the invasive VNS study by Sun et al. (2017b).

## Conclusion

While tVNS did not show similar results in enhancing cognitive performance as invasive VNS, reduction of N2 peak amplitude in NoGo-condition, along with an uncompromised response inhibition performance was observed. Reduced amplitude of N2 indicates less cognitive control resources were needed to maintain the same level of performance in healthy subjects. This suggests tVNS enhances cognitive control-related neural processes. In more general terms this study provides novel ERP evidence for tVNS influencing brain physiology tightly linked with cognitive operations. While caution is warranted, the results of this study point to future potential for noninvasively modulating neural circuits underlying cognitive control. Given non-invasive neuromodulation techniques have a huge potential as a safe and feasible treatment option, future studies are warranted to reproduce these results and to extend studies to subjects with impaired or compromised cognitive functions.

## Data Availability Statement

The datasets presented in this article are not readily available because of the hospital policy. All data collected for the purpose of the current study is stored with respect to the participants’ privacy and accessible to researchers involved in the study. Requests to access the datasets should be directed to kaisa.hartikainen@live.com.

## Ethics Statement

The study involving human participants was reviewed and approved by the Ethical Committee of the Pirkanmaa Hospital District. The patients/participants provided their written informed consent to participate in this study.

## Author Contributions

MP and LF were responsible for the recruitment of subjects, data collection, and analysis. They contributed equally to writing the manuscript. JP was responsible for the statistical analysis and participated in writing. Principal Investigator, KH was responsible for the experimental design and supervision of the data collection, analysis, interpretation of results, and writing. All authors contributed to the article and approved the submitted version.

## Conflict of Interest

The authors declare that the research was conducted in the absence of any commercial or financial relationships that could be construed as a potential conflict of interest.

## References

[B1] Alvarez J. A. Emory E. (2006). Executive function and the frontal lobes: a meta-analytic review. Neuropsychol. Rev. 16, 17–42. 10.1007/s11065-006-9002-x 16794878

[B2] Arnsten A. F. (2000). Through the looking glass: differential noradenergic modulation of prefrontal cortical function. Neural Plast. 7, 133–146. 10.1155/np.2000.133 10709220 PMC2565372

[B3] Arnsten A. F. (2011). Catecholamine influences on dorsolateral prefrontal cortical networks. Biol. Psychiatry 69, e89–e99. 10.1016/j.biopsych.2011.01.027 21489408 PMC3145207

[B4] Aston-Jones G. Cohen J. D. (2005). An integrative theory of locus coeruleus-norepinephrine function: adaptive gain and optimal performance. Annu. Rev. Neurosci. 28, 403–450. 10.1146/annurev.neuro.28.061604.135709 16022602

[B5] Back-Madruga C. Boone K. B. Briere J. Cummings J. McPherson S. Fairbanks L.. (2002). Functional ability in executive variant Alzheimer’s disease and typical Alzheimer’s disease. Clin. Neuropsychol. 16, 331–340. 10.1076/clin.16.3.331.13846 12607146

[B6] Badran B. W. Dowdle L. T. Mithoefer O. J. LaBate N. T. Coatsworth J. Brown J. C.. (2018a). Neurophysiologic effects of transcutaneous auricular vagus nerve stimulation (taVNS) *via* electrical stimulation of the tragus: a concurrent taVNS/fMRI study and review. Brain Stimul. 11, 492–500. 10.1016/j.brs.2017.12.009 29361441 PMC6487660

[B7] Badran B. W. Jenkins D. D. DeVries W. H. Dancy M. Summers P. M. Mappin G. M.. (2018b). Transcutaneous auricular vagus nerve stimulation (taVNS) for improving oromotor function in newborns. Brain Stimul. 11, 1198–1200. 10.1016/j.brs.2018.06.009 30146041 PMC6536126

[B8] Bartels C. Wegrzyn M. Wiedl A. Ackermann V. Ehrenreich H. (2010). Practice effects in healthy adults: a longitudinal study on frequent repetitive cognitive testing. BMC Neurosci. 11:118. 10.1186/1471-2202-11-118 20846444 PMC2955045

[B9] Bates D. Mächler M. Bolker B. Walker S. (2015). Fitting linear mixed-effects models using lme4. J. Stat. Softw. 67, 1–48. 10.18637/jss.v067.i01

[B10] Beck A. T. Steer R. A. Brown G. K. (1996). Manual for the Beck Depression Inventory-II (Finnish version). San Antonio, TX: The Psychological Corporation.

[B11] Berridge C. W. Spencer R. C. (2016). Differential cognitive actions of norepinephrine α2 and α1 receptor signaling in the prefrontal cortex. Brain Res. 1641, 189–196. 10.1016/j.brainres.2015.11.024 26592951 PMC4876052

[B12] Beste C. Steenbergen L. Sellaro R. Grigoriadou S. Zhang R. Chmielewski W.. (2016). Effects of concomitant stimulation of the gabaergic and norepinephrine system on inhibitory control—a study using transcutaneous vagus nerve stimulation. Brain Stimul. 9, 811–818. 10.1016/j.brs.2016.07.004 27522167

[B13] Boly M. Garrido M. I. Gosseries O. Bruno M.-A. Boveroux P. Schnakers C.. (2011). Preserved feedforward but impaired top-down processes in the vegetative state. Science 332, 858–862. 10.1126/science.1202043 21566197

[B14] Boudewyn M. A. Luck S. J. Farrens J. L. Kappenman E. S. (2018). How many trials does it take to get a significant ERP effect? It depends. Psychophysiology 55:e13049. 10.1111/psyp.13049 29266241 PMC5940511

[B15] Braver T. S. Barch D. M. Gray J. R. Molfese D. L. Snyder A. (2001). Anterior cingulate cortex and response conflict: effects of frequency, inhibition and errors. Cereb. Cortex 11, 825–836. 10.1093/cercor/11.9.825 11532888

[B16] Burger A. M. Van der Does W. Brosschot J. F. Verkuil B. (2020). From ear to eye? no effect of transcutaneous vagus nerve stimulation on human pupil dilation: a report of three studies. Biol. Psychol. 152:107863. 10.1016/j.biopsycho.2020.107863 32050095

[B17] Butt M. F. Albusoda A. Farmer A. D. Aziz Q. (2019). The anatomical basis for transcutaneous auricular vagus nerve stimulation. J. Anat. 236, 588–611. 10.1111/joa.13122 31742681 PMC7083568

[B18] Capone F. Assenza G. Di Pino G. Musumeci G. Ranieri F. Barbato C.. (2015). The effect of transcutaneous vagus nerve stimulation on cortical excitability. J. Neural Transm. 122, 679–685. 10.1007/s00702-014-1299-7 25182412

[B19] Chaytor N. Schmitter-Edgecombe M. (2003). The ecological validity of neuropsychological tests: a review of the literature on everyday cognitive skills. Neuropsychol. Rev. 13, 181–197. 10.1023/b:nerv.0000009483.91468.fb 15000225

[B20] Clarke H. F. Walker S. C. Dalley J. W. Robbins T. W. Roberts A. C. (2007). Cognitive inflexibility after prefrontal serotonin depletion is behaviorally and neurochemically specific. Cereb. Cortex 17, 18–27. 10.1093/cercor/bhj120 16481566

[B21] Colzato L. Beste C. (2020). A literature review on the neurophysiological underpinnings and cognitive effects of transcutaneous vagus nerve stimulation: challenges and future directions. J. Neurophysiol. 123, 1739–1755. 10.1152/jn.00057.2020 32208895

[B22] De Rover M. Brown S. B. Band G. P. Giltay E. J. van Noorden M. S. van der Wee N. J.. (2015). Beta receptor-mediated modulation of the oddball P3 but not error-related ERP components in humans. Psychopharmacology 232, 3161–3172. 10.1007/s00213-015-3966-2 26138780 PMC4534504

[B23] De Taeye L. Vonck K. Bochove M. V. Boon P. Roost D. V. Mollet L.. (2014). The P3 event-related potential is a biomarker for the efficacy of vagus nerve stimulation in patients with epilepsy. Neurotherapeutics 11, 612–622. 10.1007/s13311-014-0272-3 24711167 PMC4121454

[B24] Deligkaris P. Panagopoulou E. Montgomery A. J. Masoura E. (2014). Job burnout and cognitive functioning: a systematic review. Work Stress 28, 107–123. 10.1016/j.burn.2014.04.003

[B25] Diamond A. (2013). Executive functions. Annu. Rev. Psychol. 64, 135–168. 10.1146/annurev-psych-113011-143750 23020641 PMC4084861

[B26] Dietrich S. Smith J. Scherzinger C. Hofmann-Prei K. ß. Freitag T. Eisenkolb A.. (2008). A novel transcutaneous vagus nerve stimulation leads to brainstem and cerebral activations measured by functional MRI. Biomed. Eng. 53, 104–111. 10.1515/bmt.2008.022 18601618

[B27] Dixon P. (2008). Models of accuracy in repeated-measures design. J. Mem. Lang. 59, 447–456. 10.1016/j.jml.2007.11.004

[B28] Donkers F. C. van Boxtel G. J. (2004). The N2 in go/no-go tasks reflects conflict monitoring not response inhibition. Brain and Cognition 56, 165–176. 10.1016/j.bandc.2004.04.005 15518933

[B29] Dorr A. E. Debonnel G. (2006). Effect of vagus nerve stimulation on serotonergic and noradrenergic transmission. J. Pharmacol. Exp. Ther. 318, 890–898. 10.1124/jpet.106.104166 16690723

[B30] Downar J. Crawley A. P. Mikulis D. J. Davis K. D. (2000). A multimodal cortical network for the detection of changes in the sensory environment. Nat. Neurosci. 3, 277–283. 10.1038/72991 10700261

[B31] Dubreuil-Vall L. Chau P. Ruffini G. Widge A. S. Camprodon J. A. (2019). tDCS to the left DLPFC modulates cognitive and physiological correlates of executive function in a state-dependent manner. Brain Stimul. 12, 1456–1463. 10.1016/j.brs.2019.06.006 31221553 PMC6851462

[B32] Duke A. A. Bègue L. Bell R. Eisenlohr-Moul T. (2013). Revisiting the serotonin-aggression relation in humans: a meta-analysis. Psychol. Bull. 139, 1148–1172. 10.1037/a0031544 23379963 PMC3718863

[B33] Ellrich J. (2019). Transcutaneous auricular vagus nerve stimulation. J. Clin. Neurophysiol. 36, 437–442. 10.1097/WNP.0000000000000576 31688327

[B34] Englot D. J. Rolston J. D. Wright C. W. Hassnain K. H. Chang E. F. (2015). Rates and predictors of seizure freedom with vagus nerve stimulation for intractable epilepsy. Neurosurgery 79, 345–353. 10.1227/NEU.0000000000001165 26645965 PMC4884552

[B35] Erkkilä M. Peräkylä J. Hartikainen K. M. (2018). Executive functions and emotion-attention interaction in assessment of brain health: reliability of repeated testing with executive RT test and correlation with BRIEF-A questionnaire. Front. Psychol. 9:2556. 10.3389/fpsyg.2018.02556 30618977 PMC6297677

[B36] Falkenstein M. Hoormann J. Hohnsbein J. (1999). ERP components in Go/Nogo tasks and their relation to inhibition. Acta Psychol. 101, 267–291. 10.1016/s0001-6918(99)00008-6 10344188

[B37] Fanselow E. E. (2012). Central mechanisms of cranial nerve stimulation for epilepsy. Surg. Neurol. Int. 3, 247–254. 10.4103/2152-7806.103014 23230529 PMC3514917

[B38] Fischer R. Ventura-Bort C. Hamm A. Weymar M. (2018). Transcutaneous vagus nerve stimulation (tVNS) enhances conflict-triggered adjustment of cognitive control. Cogn. Affect. Behav. Neurosci. 18, 680–693. 10.3758/s13415-018-0596-2 29693214

[B39] Folstein J. R. Van Petten C. (2008). Influence of cognitive control and mismatch on the N2 component of the ERP: a review. Psychophysiology 45, 152–170. 10.1111/j.1469-8986.2007.00602.x 17850238 PMC2365910

[B40] Frangos E. Komisaruk B. R. (2017). Access to vagal projections *via* cutaneous electrical stimulation of the neck: fMRI evidence in healthy humans. Brain Stimul. 10, 19–27. 10.1016/j.brs.2016.10.008 28104084

[B41] Grimonprez A. Raedt R. Baeken C. Boon P. Vonck K. (2015a). The antidepressant mechanism of action of vagus nerve stimulation: evidence from preclinical studies. Neurosci. Biobehav. Rev. 56, 26–34. 10.1016/j.neubiorev.2015.06.019 26116875

[B42] Grimonprez A. Raedt R. Portelli J. Dauwe I. Larsen L. E. Bouckaert C.. (2015b). The antidepressant-like effect of vagus nerve stimulation is mediated through the locus coeruleus. J. Psychiatr. Res. 68, 1–7. 10.1016/j.jpsychires.2015.05.002 26228393

[B43] Haatveit B. Vaskinn A. Sundet K. S. Jensen J. Andreassen O. A. Melle I.. (2015). Stability of executive functions in first episode psychosis: 1 year follow up study. Psychiatry Res. 228, 475–481. 10.1016/j.psychres.2015.05.060 26165960

[B44] Hanna-Pladdy B. (2007). Dysexecutive syndromes in neurologic disease. J. Neurol. Phys. Ther. 31, 119–127. 10.1097/npt.0b013e31814a63c2 18025957

[B45] Hartikainen K. M. Ogawa K. H. Knight R. T. (2000). Transient interference of right hemispheric function due to automatic emotional processing. Neuropsychologia 38, 1576–1580. 10.1016/s0028-3932(00)00072-5 11074080

[B46] Hartikainen K. M. Ogawa K. H. Knight R. T. (2010a). Trees over forest: unpleasant stimuli compete for attention with global features. Neuroreport 21, 344–348. 10.1097/wnr.0b013e328336eeb3 20168261 PMC2922681

[B47] Hartikainen K. M. Ogawa K. H. Soltani M. Knight R. T. (2007). Emotionally arousing stimuli compete for attention with left hemispace. Neuroreport 18, 1929–1933. 10.1097/wnr.0b013e3282f1ca18 18007189

[B48] Hartikainen K. M. Siiskonen A. R. Ogawa K. H. (2012). Threat interferes with response inhibition. Neuroreport 23, 447–450. 10.1097/wnr.0b013e3283531e74 22494999

[B49] Hartikainen K. M. Sun L. Polvivaara M. Brause M. Lehtimäki K. Haapasalo J.. (2014). Immediate effects of deep brain stimulation of anterior thalamic nuclei on executive functions and emotion-attention interaction in humans. J. Clin. Exp. Neuropsychol. 36, 540–550. 10.1080/13803395.2014.913554 24839985 PMC4066928

[B50] Hartikainen K. M. Waljas M. Isoviita T. Dastidar P. Liimatainen S. Solbakk A.-K.. (2010b). Persistent symptoms in mild to moderate traumatic brain injury associated with executive dysfunction. J. Clin. Exp. Neuropsychol. 32, 767–774. 10.1080/13803390903521000 20198531

[B51] Hermans L. Leunissen I. Pauwels L. Cuypers K. Peeters R. Puts N. A.. (2018). Brain GABA levels are associated with inhibitory control deficits in older adults. J. Neurosci. 38, 7844–7851. 10.1523/JNEUROSCI.0760-18.2018 30064995 PMC6125814

[B52] Hirata A. Aguilar J. Castro-Alamancos M. A. (2006). Noradrenergic activation amplifies bottom-up and top-down signal-to-noise ratios in sensory thalamus. J. Neurosci. 26, 4426–4436. 10.1523/JNEUROSCI.5298-05.2006 16624962 PMC6674001

[B53] Holmes G. L. (2015). Cognitive impairment in epilepsy: the role of network abnormalities. Epileptic Disord. 17, 101–116. 10.1684/epd.2015.0739 25905906 PMC5410366

[B54] Jacobs H. I. Leritz E. C. Williams V. J. Van Boxtel M. P. van der Elst W. Jolles J.. (2013). Association between white matter microstructure, executive functions, and processing speed in older adults: the impact of vascular health. Hum. Brain Mapp. 34, 77–95. 10.1002/hbm.21412 21954054 PMC3830829

[B55] Jacobs H. I. Riphagen J. M. Razat C. M. Wiese S. Sack A. T. (2015). Transcutaneous vagus nerve stimulation boosts associative memory in older individuals. Neurobiol. Aging 36, 1860–1867. 10.1016/j.neurobiolaging.2015.02.023 25805212

[B56] Jaeger T. F. (2008). Categorical data analysis: away from ANOVAs (transformation or not) and towards logit mixed models. J. Mem. Lang. 59, 434–446. 10.1016/j.jml.2007.11.007 19884961 PMC2613284

[B57] Jodoin V. D. Lespérance P. Nguyen D. K. Fournier-Gosselin M.-P. Richer F. (2015). Effects of vagus nerve stimulation on pupillary function. Int. J. Psychophysiol. 98, 455–459. 10.1016/j.ijpsycho.2015.10.001 26437126

[B58] Jongkees B. J. Immink M. A. Finisguerra A. Colzato L. S. (2018). Transcutaneous vagus nerve stimulation (tVNS) enhances response selection during sequential action. Front. Psychol. 9:1159. 10.3389/fpsyg.2018.01159 30034357 PMC6043681

[B59] Kalia M. Sullivan M. J. (1982). Brainstem projections of sensory and motor components of the vagus nerve in the rat. J. Comp. Neurol. 211, 248–265. 10.1002/cne.902110304 7174893

[B60] Keute M. Demirezen M. Graf A. Mueller N. G. Zaehle T. (2019). No modulation of pupil size and event-related pupil response by transcutaneous auricular vagus nerve stimulation (taVNS). Sci. Rep. 9:11452. 10.1038/s41598-019-47961-4 31391505 PMC6685960

[B61] Kok A. (2001). On the utility of P3 amplitude as a measure of processing capacity. Psychophysiology 38, 557–577. 10.1017/s0048577201990559 11352145

[B62] Kopp B. Mattler U. Goertz R. Rist F. (1996). N2, P3 and the lateralized readiness potential in a nogo task involving selective response priming. Electroencephalogr. Clin. Neurophysiol. 99, 19–27. 10.1016/0921-884x(96)95617-9 8758967

[B63] Kouider S. Stahlhut C. Gelskov S. V. Barbosa L. S. Dutat M. de Gardelle V.. (2013). A neural marker of perceptual consciousness in infants. Science 340, 376–380. 10.1126/science.1232509 23599498

[B64] Kraiser S. Weiss O. Hill H. Markela-Lerenc J. Kiefer M. Weisbrod M. (2006). N2 event-related potential correlates of response inhibition in an auditory Go/Nogo task. Int. J. Psychophysiol. 61, 279–282. 10.1016/j.ijpsycho.2005.09.006 16298004

[B65] Kraus T. Hösl K. Kiess O. Schanze A. Kornhuber J. Foster C. (2007). BOLD fMRI deactivation of limbic and temporal brain structures and mood enhancing effect by transcutaneous vagus nerve stimulation. J. Neural Transm. 114, 1485–1493. 10.1007/s00702-007-0755-z 17564758

[B66] Kraus T. Kiess O. Hösl K. Terekhin P. Kornhuber J. Forster C. (2013). CNS BOLD fMRI effects of sham-controlled transcutaneous electrical nerve stimulation in the left outer auditory canal—a pilot study. Brain Stimul. 6, 798–804. 10.1016/j.brs.2013.01.011 23453934

[B67] Kuusinen V. Cesnaite E. Peräkylä J. Ogawa K. H. Hartikainen K. M. (2018). Orbitofrontal lesion alters brain dynamics of emotion-attention and emotion-cognitive control interaction in humans. Front. Hum. Neurosci. 12:437. 10.3389/fnhum.2018.00437 30443211 PMC6221981

[B68] Løvstad M. Funderud I. Endestad T. Due T. ønnessen P. Meling T. R.. (2012a). Executive functions after orbital or lateral prefrontal lesions: neuropsychological profiles and self-reported executive functions in everyday living. Brain Inj. 26, 1586–1598. 10.3109/02699052.2012.698787 22731818 PMC4090100

[B69] Løvstad M. Funderud I. Meling T. Krämer U. M. Voytek B. Due T.. (2012b). Anterior cingulate cortex and cognitive control: neuropsychological and electrophysiological findings in two patients with lesions to dorsomedial prefrontal cortex. Brain Cogn. 80, 237–249. 10.1016/j.bandc.2012.07.008 22935543 PMC4067454

[B70] Lapenta O. M. Di Sierve K. de Macedo E. C. Fregni F. Boggio P. S. (2014). Transcranial direct current stimulation modulates ERP-indexed inhibitory control and reduces food consumption. Appetite 83, 42–48. 10.1016/j.appet.2014.08.005 25128836

[B71] Lavric A. Pizzagalli D. A. Forstmeier S. (2004). When “go” and “nogo” are equally frequent: ERP components and cortical tomography. Eur. J. Neurosci. 20, 2483–2488. 10.1111/j.1460-9568.2004.03683.x 15525290

[B72] Lawrence M. A. (2016). ez: Easy Analysis and Visualization of Factorial Experiments. R package version 4.4–0. 2. November [Online]. Available online at: https://github.com/mike-lawrence/ez. Accessed July 24, 2020.

[B73] Leventhal A. G. Wang Y. Pu M. Zhou Y. Ma Y. (2003). GABA and its agonists improved visual cortical function in senescent monkeys. Science 300, 812–815. 10.1126/science.1082874 12730605

[B74] Lezak M. D. (1982). The problem of assessing executive functions. Int. J. Psychol. 17, 281–297. 10.1080/00207598208247445

[B75] Liimatainen J. Peräkylä J. Järvelä K. Sisto T. Yli-Hankala A. Hartikainen K. M. (2016). Improved cognitive flexibility after aortic valve replacement surgery. Int. Cardiovasc. Thoracic Surg. 23, 630–636. 10.1093/icvts/ivw170 27245618

[B76] Llanos F. McHaney J. R. Schuerman W. L. Yi H. G. Leonard M. K. Chandrasekaran B. (2020). Non-invasive peripheral nerve stimulation selectively enhances speech category learning in adults. NPJ Sci. Learn. 5:12. 10.1038/s41539-020-0070-0 32802406 PMC7410845

[B77] Luck S. J. (2005). An Introduction to the Event-related Potential Technique. Cambridge, MA: The MIT PRESS.

[B78] Mäki-Marttunen V. Kuusinen V. Brause M. Peräkylä J. Polvivaara M. Ribeiro R. D.. (2015). Enhanced attention capture by emotional stimuli in mild traumatic brain injury. J. Neurotrauma 32, 272–279. 10.1089/neu.2014.3557 25274125 PMC4321980

[B79] Mäki-Marttunen V. Kuusinen V. Peräkylä J. Ogawa K. H. Brause M. Brander A.. (2017). Greater attention to task-relevant threat due to orbitofrontal lesion. J. Neurotrauma 34, 400–413. 10.1089/neu.2015.4390 27502875

[B80] Mair R. D. Zhang Y. Bailey K. R. Toupin M. M. Mair R. G. (2005). Effects of clonidine in the locus coeruleus on prefrontal- and hippocampal-dependent measures of attention and memory in the rat. Psychopharmacology 181, 280–288. 10.1007/s00213-005-2263-x 15830223

[B81] Manta S. El Mansari M. Debonnel G. Blier P. (2013). Electrophysiological and neurochemical effects of long-term vagus nerve stimulation on the rat monoaminergic systems. Int. J. Neuropsychopharmacol. 16, 459–470. 10.1017/s1461145712000387 22717062

[B82] Marshall G. A. Rentz D. M. Frey M. T. Locascio J. J. Johnson K. A. Sperling R. A. (2011). Executive function and instrumental activities of daily living in MCI and AD. Alzheimers Dement. 7, 300–308. 10.1016/j.jalz.2010.04.005 21575871 PMC3096844

[B83] Miyake A. Friedman N. P. Emerson M. J. Witzki A. H. Howerter A. Wager T. D. (2000). The unity and diversity of executive functions and their contributions to complex “Frontal Lobe” tasks: a latent variable analysis. Cogn. Psychol. 41, 49–100. 10.1006/cogp.1999.0734 10945922

[B84] Neuhaus A. H. Luborzewski A. Rentzsch J. Brakemeier E. L. Opgen-Rhein C. Gallinat J.. (2007). P300 is enhanced in responders to vagus nerve stimulation for treatment of major depressive disorder. J. Affect. Disord. 100, 123–128. 10.1016/j.jad.2006.10.005 17098290

[B85] Nieuwenhuis S. de Geus E. J. Aston-Jones G. (2011). The anatomical and functional relationship between the P3 and autonomic components of the orienting response. Psychophysiology 48, 162–175. 10.1111/j.1469-8986.2010.01057.x 20557480 PMC3797154

[B86] Nieuwenhuis S. Yeung N. van den Wildenberg W. Ridderinkhof K. R. (2003). Electrophysiological correlates of anterior cingulate function in a go/no-go task: effects of response conflict and trial type frequency. Cognit. Affect. Behav. Neurosci. 3, 17–26. 10.3758/cabn.3.1.17 12822595

[B87] O’Reardon J. P. Christancho P. Peshek A. D. (2006). Vagus nerve stimulation (VNS) and treatment of depression: to the brainstem and beyond. Psychiatry 3, 54–63. 21103178 PMC2990624

[B88] Peräkylä J. Sun L. Lehtimäki K. Peltola J. Öhman J. Möttönen T.. (2017). Causal evidence from humans for the role of mediodorsal nucleus of the thalamus in working memory. J. Cogn. Neurosci. 29, 2090–2102. 10.1162/jocn_a_01176 28777058

[B89] Polich J. (2007). Updating P300: an integrative theory of P3a and P3b. Clin. Neuropsychol. 118, 2128–2148. 10.1016/j.clinph.2007.04.019 17573239 PMC2715154

[B90] R Core Team R. (2019). A Language and Environment for Statistical Computing. Vienna, Austria: R Foundation for Statistical Computing [Online]. Available online at: http://www.r-project.org/index.html. Accessed July 24, 2020.

[B91] Redgrave J. Day D. Leung H. Laud P. J. Ali A. Lindert R.. (2018). Safety and tolerability of transcutaneous vagus nerve stimulation in humans; a systematic review. Brain Stimul. 11, 1225–1238. 10.1016/j.brs.2018.08.010 30217648

[B92] Riepe M. W. Riss S. Bittner D. Huber R. (2004). Screening for cognitive impairment in patients with acute stroke. Dement. Geriatr. Cogn. Disord. 17, 49–53. 10.1159/000074082 14560065

[B93] Rogers M. A. Kasai K. Koji M. Fukuda R. Iwanami A. Nakagome K.. (2004). Executive and prefrontal dysfunction in unipolar depression: a review of neuropsychological and imaging evidence. Neurosci. Res. 50, 1–11. 10.1016/j.neures.2004.05.003 15288493

[B94] Roosevelt R. W. Smith D. C. Clough R. W. Jensen R. A. Browning R. A. (2006). Increased extracellular concentrations of norepinephrine in cortex and hippocampus following vagus nerve stimulation in the rat. Brain Res. 1119, 124–132. 10.1016/j.brainres.2006.08.048 16962076 PMC1751174

[B95] Roth R. M. Isquith P. K. Gioia G. A. (2005). Behavior Rating Inventory of Executive Function-Adult Version (BRIEF-A). North Florida Avenue, Lutz, Florida: Psychological Assessment Resources, Inc.

[B96] Schevernels H. van Bochove M. De Taeye L. Bombeke K. Vonck K. Van Roost D.. (2016). The effect of vagus nerve stimulation on response inhibition. Epilepsy Behav. 64, 171–179. 10.1016/j.yebeh.2016.09.014 27743550

[B97] Sellaro R. van Leusden J. W. Tona K.-D. Verkuil B. Nieuwenhuis S. Colzato L. S. (2015). Transcutaneous vagus nerve stimulation enhances post-error slowing. J. Cogn. Neurosci. 27, 2126–2132. 10.1162/jocn_a_00851 26226074

[B98] Steenbergen L. Colzato L. S. Maraver M. J. (2020). Vagal signaling and the somatic marker hypothesis: the effect of transcutaneous vagal nerve stimulation on delay discounting is modulated by positive mood. Int. J. Psychophysiol. 148, 84–92. 10.1016/j.ijpsycho.2019.10.010 31734442

[B99] Sun L. Peräkylä J. Hartikainen K. M. (2017a). Frontal alpha asymmetry, a potential biomarker for the effect of neuromodulation on brain’s affective circuitry—preliminary evidence from a deep brain stimulation study. Front. Hum. Neurosci. 11:584. 10.3389/fnhum.2017.00584 29255409 PMC5722792

[B100] Sun L. Peräkylä J. Holm K. Haapasalo J. Lehtimäki K. Ogawa K. H.. (2017b). Vagus nerve stimulation improves working memory performance. J. Clin. Exp. Neuropsychol. 39, 954–964. 10.1080/13803395.2017.1285869 28492363

[B101] Sun L. Peräkylä J. Kovalainen A. Ogawa K. H. Karhunen P. J. Hartikainen K. M. (2016). Human brain reacts to transcranial extraocular light. PLoS One 11:e0149525. 10.1371/journal.pone.0149525 26910350 PMC4767140

[B102] Sun L. Peräkylä J. Polvivaara M. Öhman J. Peltola J. Lehtimäki K.. (2015). Human anterior thalamic nuclei are involved in emotion-attention interaction. Neuropsychologia 78, 88–94. 10.1016/j.neuropsychologia.2015.10.001 26440152

[B103] Van Bockstaele E. J. Telegan P. Peoples J. (1999). Efferent projections of the nucleus of the solitary tract to peri-locus coeruleus dendrites in rat brain: evidence for a monosynaptic pathway. J. Comp. Neurol. 412, 410–428. 10.1002/(sici)1096-9861(19990927)412:3<410::aid-cne3>3.0.co;2-f 10441230

[B104] Ventura-Bort C. Wirkner J. Genheimer H. Wendth J. Hamm A. O. Weymar M. (2018). Effects of transcutaneous vagus nerve stimulation (tVNS) on the P300 and alpha-amylase level: a pilot study. Front. Hum. Neurosci. 12:202. 10.3389/fnhum.2018.00202 29977196 PMC6021745

[B105] Verdejo-García A. Pérez-García M. (2007). Ecological assessment of executive functions in substance dependent individuals. Drug Alcohol Depend. 90, 48–55. 10.1016/j.drugalcdep.2007.02.010 17382487

[B106] Wang M. Ramos B. P. Paspalas C. D. Shu Y. Simen A. Duque A.. (2007). α2A-adrenoceptors strengthen working memory networks by inhibiting cAMP-HCN channel signaling in prefrontal cortex. Cell 129, 397–410. 10.1016/j.cell.2007.03.015 17448997

[B108] Warren C. V. Maraver M. J. de Luca A. Kopp B. (2020). The effect of transcutaneous auricular vagal nerve stimulation (taVNS) on P3 event-related potentials during a bayesian oddball task. Brain Sci. 10:404. 10.3390/brainsci10060404 32630571 PMC7349824

[B107] Warren C. M. Tona K. D. Ouwerker L. van Paridon J. V. Poletiek F. van Steenbergen H.. (2019). The neuromodulatory and hormonal effects of transcutaneous vagus nerve stimulation as evidenced by salivary alpha amylase, salivary cortisol, pupil diameter, and the P3 event-related potential. Brain Stimul. 12, 635–642. 10.1016/j.brs.2018.12.224 30591360

[B109] Watanabe Y. Funahashi S. (2012). Thalamic mediodorsal nucleus and working memory. Neurosci. Biobehav. Rev. 36, 134–142. 10.1016/j.neubiorev.2011.05.003 21605592

[B110] Woodward D. J. Moises H. C. Waterhouse B. D. Hoffer B. J. Freedman R. (1979). Modulatory actions of norepinephrine in the central nervous system. Fed. Proc. 38, 2109–2116. 446766

[B111] Yeo I.-K. Johnson R. A. (2000). A new family of power transformations to improve normality or symmetry. Biometrika 87, 954–959. 10.1093/biomet/87.4.954

[B112] Yerkes R. M. Dodson J. D. (1908). The relation of strength of stimulus to rapidity of habit-formation. J. Comp. Neurol. Psychol. 18, 459–482. 10.1002/cne.920180503

